# Diffusion tensor imaging (DTI) of human lower leg muscles: correlation between DTI parameters and muscle power with different ankle positions

**DOI:** 10.1007/s11604-022-01274-1

**Published:** 2022-04-09

**Authors:** Shoichiro Takao, Maho Kaneda, Mihoko Sasahara, Suzuka Takayama, Yoshitaka Matsumura, Tetsuya Okahisa, Tsuyoshi Goto, Nori Sato, Shinsuke Katoh, Masafumi Harada, Junji Ueno

**Affiliations:** 1 Department of Diagnostic Radiology, Graduate School of Health Sciences, Tokushima University, 3-18-15, Kuramoto-Cho, Tokushima, 770-8509 Japan; 2 Department of Radiology, Grandsoul Nara, Nara, Japan; 3 Department of Radiology, Tokushima University, Tokushima, Japan; 4 Department of Radiology, Rakuwakai Otowa Hospital, Kyoto, Japan; 5 Department of Radiological Technology, Tokushima Bunri University, Kagawa, Japan; 6 Graduate School of Health Sciences, Tokushima University, Tokushima, Japan; 7 Rehabilitation Center, Tokushima University Hospital, Tokushima, Japan; 8 Department of Rehabilitation, Japanese Red Cross Tokushima Hinomine Rehabilitation Center, Tokushima, Japan; 9 Department of Radiology, Kitajima Taoka Hospital, Tokushima, Japan

**Keywords:** Diffusion tensor imaging, Lower leg muscles, Muscle contraction, Muscle power

## Abstract

**Purpose:**

To compare diffusion tensor imaging (DTI) parameters in healthy adult human lower leg muscles and to determine the correlation between DTI parameters and muscle power measurements among different types of muscle contraction.

**Materials and methods:**

DTI measurements of the unilateral lower leg muscles having three different types of contraction (non-contraction state, isometric contraction, and soleus shortening) were obtained from 10 healthy adults using a 3-T MRI scanner. DTI parameters (λ_1_, λ_2_, λ_3_, mean diffusivity, and fractional anisotropy) were calculated. The values of the DTI parameters and correlation between the DTI parameters and muscle power measurements (maximum power and maximum amount of work) obtained from a dynamometer were statistically compared among the different types of contraction. Intra- and inter-class correlation coefficients were calculated for analysis of reproducibility.

**Results:**

The λ_1_, λ_2_, λ_3,_ and mean diffusivity of the soleus muscle are significantly lower in the non-contraction state as compared with isometric contraction and soleus shortening (*p* < 0.05). A positive correlation of the soleus muscle in the non-contraction state was seen between the maximum power and the λ_1_, λ_2,_ and mean diffusivity. There was a positive correlation between the maximum amount of work and fractional anisotropy in the non-contraction state for the soleus muscle. A negative correlation for the tibialis anterior muscle in the non-contraction state was seen between the maximum amount of work and fractional anisotropy. Overall reproducibility of the DTI parameters was excellent.

**Conclusions:**

DTI parameters were significantly changed depending on the ankle joint position and type of muscle contraction.

**Supplementary Information:**

The online version contains supplementary material available at 10.1007/s11604-022-01274-1.

## Introduction

With the recent development of magnetic resonance imaging (MRI) and its applications, diffusion weighted MR imaging (DW MRI) can be obtained using various commercial MRI scanners. DW MRI detects the random motion of molecules, called Brownian motion, at a microscopic level [1]. With DW MRI, the displacement distribution of water molecules within the imaging voxels can be observed, and, thus, may provide unique clues to the structure and architectural organization of tissues [1]. Since water diffusion is a three-dimensional process, anisotropy of water diffusion may exist. This anisotropy may result from the presence of an obstacle which limits the molecular movement in some directions [1]. Diffusion tensor imaging (DTI) can be achieved by measuring the apparent diffusion coefficient (ADC) in at least six independent directions to quantify the directional anisotropy of the diffusion [2]. For quantitative analysis of DTI, several DTI parameters (λ_1_, λ_2_, λ_3_, mean diffusivity, and fractional anisotropy) can be calculated. The λ_1_, λ_2,_ and λ_3_ are called “eigenvalues” which express the diffusivity of three orthogonal diffusion directions. Assuming that molecular diffusion is ellipsoid with anisotropy, λ_1_ (principal eigenvalues) represents the direction with highest diffusion, and λ_2_ and λ_3_ represent diffusions with two orthogonal directions perpendicular to λ_1_. The relationship among these three eigenvalues is as follows: λ_1_ ≥ λ_2_ ≥ λ_3_. The mean diffusivity (MD) is a dimensionless index which describes the directional average of diffusion in the tissue [2]. Fractional anisotropy (FA) is a dimensionless index of the anisotropy of diffusion. If the molecular diffusion is isotropic, the FA value equals 0, and if the diffusion is cylindrically symmetric anisotropic, the FA value approaches 1 [2]. The MD and FA can be calculated using the following formulae:

$${\text{MD}}\, = \,\frac{{\lambda_{1} + \lambda_{2} + \lambda_{3} }}{3}$$

$${\text{FA }} = \, \sqrt{\frac{3}{2}} \frac{{\sqrt {\left( {\lambda_{1} - {\text{MD}}} \right)^{2} + \left( {\lambda_{2} - {\text{MD}}} \right)^{2} + \left( {\lambda_{3} - {\text{MD}}} \right)^{2} } }}{{\sqrt {\lambda_{1}^{2} + \lambda_{2}^{2} + \lambda_{3}^{2} } }}$$

The microstructure of skeletal muscle can be evaluated quantitatively when analyzed by DTI. Assuming that skeletal muscle consists of highly ordered, elongated cylindrical muscle fibers, the λ_1_ direction represents diffusive sampling to the long axis of the muscle fibers, the λ_2_ direction represents pathways along sheets of individual muscle fibers within the endomysium, and the λ_3_ direction represents the pathway within the individual fibers [3, 4].

There have been several articles in which DTI was used to evaluate physiological conditions in healthy skeletal muscle. Scheel et al. analyzed the correlation between the DTI parameters and maximum muscle power in the soleus muscle as measured by a dynamometer, and found a negative correlation with the FA and a positive correlation with the radial diffusivity [5]. Okamoto et al. measured the DTI parameters of thigh muscle before and after hybrid training in patients with non-alcoholic fatty liver disease and found that the FA, ADC, and eigenvalues increased post training [6]. Based on these results, either the improvement or progression of muscle weakness in patients with neuromuscular disease, such as cerebral infarction or muscle dystrophy, can be evaluated objectively in a minimally invasive manner. Furthermore, muscle strength and the effect of training of athletes can be objectively evaluated with DTI. However, the values of the DTI parameters have been reported to change with demographic factors, such as gender [7] and age [8], or with transient factors, such as temperature [9], joint position, and muscle contraction status, during DTI acquisition [10, 11]. Considering these effects on the DTI parameters, the correlation between the DTI parameters and muscle power measurements might be changed if the transient factors of joint position or muscle contraction status are not uniform during the DTI acquisition. During the DTI acquisition, some degree of isometric contraction in the soleus and anterior tibialis muscles could occur when the ankle joint angle is maintained and even if the foot is fixed.

The aim of this study was to obtain the values of the DTI parameters in healthy adult human lower leg muscles and to correlate the DTI parameters and muscle power measurements among different types of muscle contraction.

## Materials and methods

### Subjects

Ten healthy adult male volunteers were recruited for this study. The mean age was 22.9 years (range 21–27), and the mean body mass index (BMI) was 20.2 (range 16.8–24.7). The inclusion criteria for all subjects were: age between 20 and 30 years, no history of lower leg muscle disease, and no current lower leg muscle symptoms. All subjects were instructed to refrain from leg exercise, such as running or long-distance walking during the 48 h prior to the MR examination and muscle power measurements. The study protocol was approved by the institutional review board (Protocol Number: 2207-3) and conformed to the Declaration of Helsinki. Written informed consent was obtained from each subject.

### MR imaging protocol

All MR images were obtained on a 3.0-Tesla scanner (Discovery MR750, GE Medical Systems, Milwaukee, Wis., USA) using an 8-channel HD TR knee PA coil. For each subject, three pairs of axial T2*-weighted images (T2*WI) and diffusion tensor imaging (DTI) of the right lower leg were obtained with three different types of muscle contraction (non-contraction, isometric contraction, and soleus shortening). The MRI acquisition parameters are shown in Table 1.

**Table 1:** MR imaging parameters

	DTI	T2*WI
Sequence	Echo planar imaging	Gradient echo
Mode	2D	2D
b-value (s/mm^2^)	600	–
MPG directions	12	–
TR/TE (ms)	2500/51.3	400/4.9
FOV (mm)	160 × 160	160 × 160
Matrix size	128 × 128	128 × 128
Flip angle (degree)	90	20
Slice thickness/gap (mm)	3/0	3/0
Number of slices	5	5
NEX	8	1
Acquisition time	4 min 23 s	0 min 46 s

*MPG* motion-probing gradient, *TR* repetition time, *TE* echo time, *FOV* field of view, and *NEX* number of excitations

For each subject, a small skin marker was placed at the level of the maximum lower leg circumference defined visually as a reference of the imaging plane. For the non-contraction state, a wooden flat plate was placed under the plantar side of the foot to fix the neutral ankle joint angle position during MRI acquisition. For the isometric contraction state, a wooden flat plate and an elastic hard ball were placed under the plantar side of the foot, and each subject was instructed to maintain the neutral ankle joint angle position during MRI acquisition. For the soleus (SOL) shortening state, the ankle joint angle was fixed at each subject’s maximum plantar flexion during MRI acquisition. The information on the angle of the ankle joint and on the contraction status of the tibialis anterior and soleus muscles is summarized at Table 2.

**Table 2:** Types of muscle contraction

	non-contraction	isometric contraction	soleus (SOL) shortening
			
angle of ankle joint	neutral position	neutral position	maximum plantar flexion
status of tibialis anterior muscle	non-contraction	isometric contraction	elongation
status of soleus muscle	non-contraction	isometric contraction	contraction with shortening

**Fig. 1:**
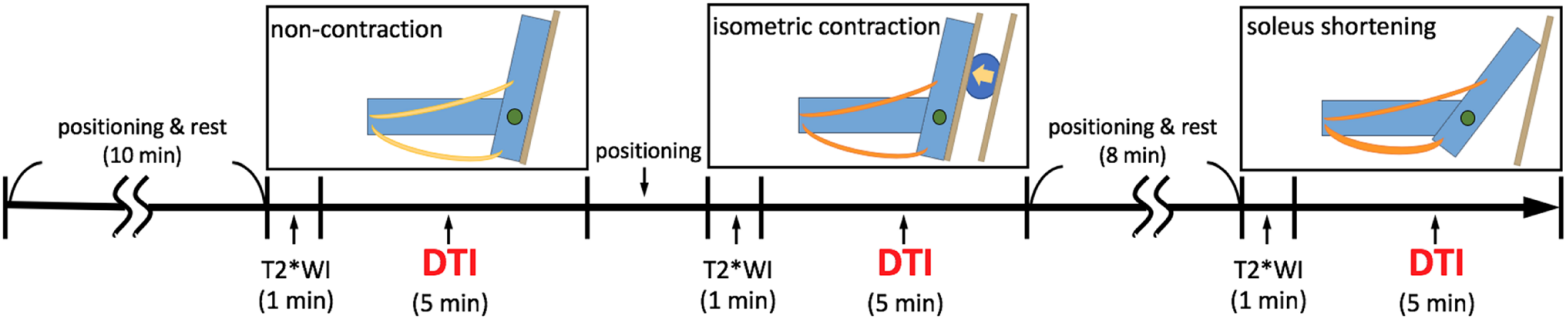
MR imaging protocol

After 10 min of positioning and rest, T2*WI and DTI with three different ankle joint angles and the muscle contraction status were obtained. For eliminating the effect of the DTI parameter change with muscle contraction, 8 min of positioning and resting time occurred between the MRI acquisition with isometric contraction and soleus shortening. MRI acquisition was conducted in the knee extension position. A schematic illustration of the MRI protocol is shown in Fig. 1.

### DTI processing and muscle segmentation

**Fig. 2:**
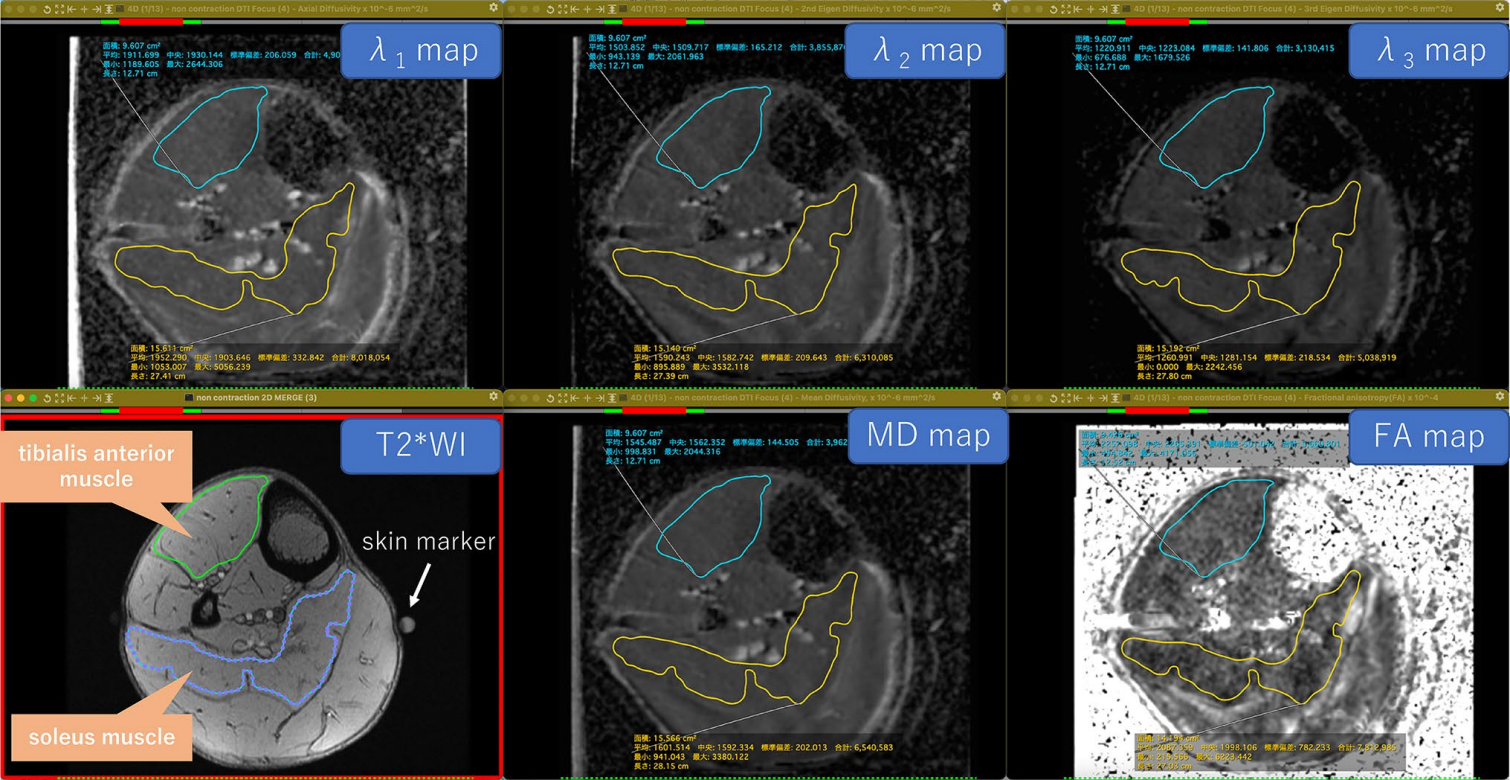
DTI images and muscle segmentation *MD* mean diffusivity, *FA* fractional anisotropy. Areas inside the green and light blue lines: ROIs of the tibialis anterior muscle Areas inside the purple dots and orange lines: ROIs of the soleus muscle

Subsequent to the MRI examination, isotropic images of each DTI were generated using software FuncTools (GE Medical Systems, Milwaukee, Wis., USA). All T2*WI and DTI were transferred in the DICOM (Digital Imaging and Communications in Medicine) format to a personal computer. The λ_1_, λ_2_, λ_3_, mean diffusivity (MD), and fractional anisotropy (FA) maps were generated from each DTI. The entire right anterior tibialis and soleus muscle at the level of maximum circumference, excluding the fascia of the muscle, were segmented from the T2*WI by drawing a polygonal region of interest (ROI), and the ROI was copied and pasted to the generated λ_1_, λ_2_, λ_3_, MD, and FA maps. Then, distinct imaging artifacts were extracted from the ROIs and any incorrect registration of muscle contours was corrected manually. To evaluate the cross-sectional area (CSA) of each muscle, segmentation was performed by tracing the fascia of the muscle. All of this DTI processing and muscle segmentation were performed using software Osirix MD version 9.5.1, Osirix Lite version. 9.0 (Pixmeo Sarl, Switzerland), and plug-in software DTIMap version 1.6 (Dequiang Qiu, Emory University). The average λ_1_, λ_2_, λ_3_, MD, and FA values of each ROI were calculated and used for analysis (Fig. 2).

### Muscle power measurements

**Fig. 3:**
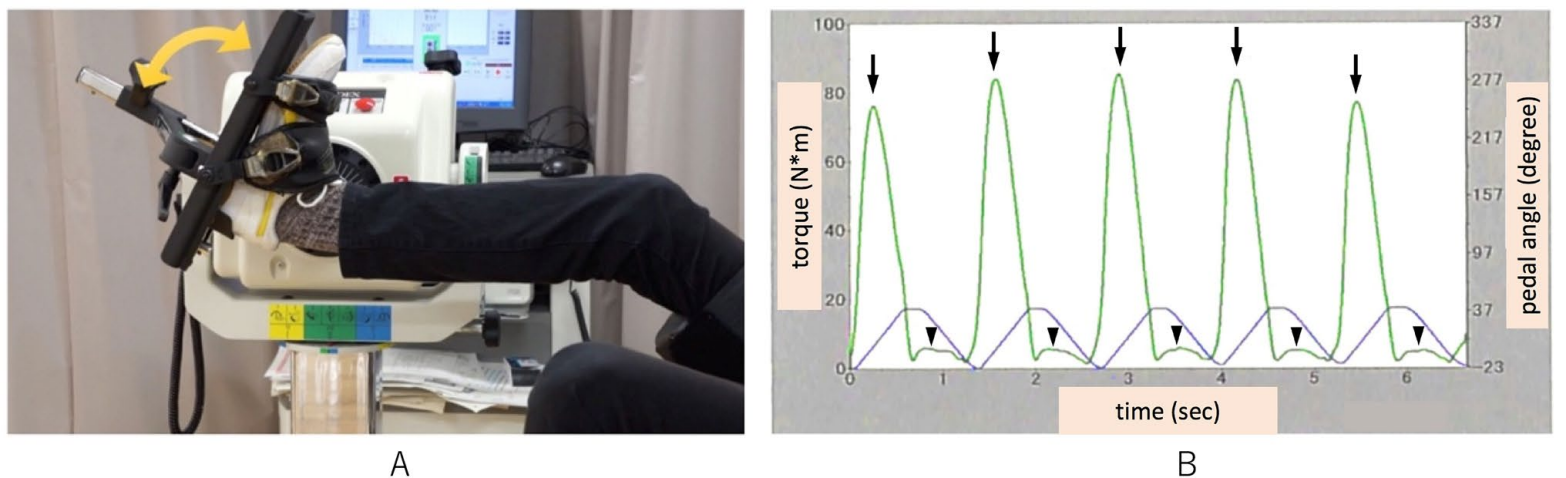
Muscle power measurement **A** Each subject performed a pair of isokinetic plantarflexions and dorsiflexions for the muscle power measurement. **B** Time–torque curve obtained from five pairs of isokinetic plantarflexions and dorsiflexions. The green curve represents the time–torque curve (arrows: peaks of soleus muscle contraction phase, arrow heads: peaks of tibialis anterior muscle contraction phase). The purple curve represents the pedal angle curve

Muscle power measurements were performed using a dynamometer (BIODEX system4, Biodex Medical Systems, NY, USA) within 3 days after the MRI. During the muscle power measurement, each subject’s body was fixed on a chair and the plantar side of the right foot was maintained on a flat pedal. Following a warm up period, each subject performed five pairs of isokinetic plantarflexion and dorsiflexion with three different angular velocities (30, 60, and 120 rad/s) (Fig. 3A). Body weight-corrected maximum torque and body weight-corrected maximum amount of work were measured from the time-torque curves (Fig. 3B). The maximum power was calculated using the following formula:

$$maximum\;power\left( W \right) = maximum{\mkern 1mu} \;torque\left( {N*m} \right) \times angular\;velocities\;\left( {rad/sec} \right)$$

The highest maximum power among the above-mentioned three different angular velocities was used for analysis.

### Statistical analysis

The values of DTI parameters and the correlation between the DTI parameters and muscle power measurements (maximum power and maximum amount of work) obtained from the dynamometer were statistically compared among the different types of contraction. Friedman’s test was used for the multiple comparison analysis. Spearman’s rank correlation coefficient was used for the correlation analysis, and the *r*_*s*_ were calculated. The strength of the correlation was defined as follows: “very weak”: 0.00 ≤| *r*_*s*_ |< 0.19, “weak”: 0.20 ≤| *r*_*s*_ |< 0.39, “moderate”: 0.40 ≤| *r*_*s*_ |< 0.59, “strong”: 0.60 ≤| *r*_*s*_ |< 0.79, and “very strong”: 0.80 ≤| *r*_*s*_ |≤ 1.00.

Inter-class and intra-class correlation coefficients (inter- and intra-ICCs) were calculated for analysis of intra- and inter-observer reproducibility for observer 1 (15 years of experience in musculoskeletal radiology) and observer 2 (2 years of experience in musculoskeletal radiology). The relative strength of agreement was defined as poor (ICC < 0.40), fair (ICC 0.40–0.59), good (ICC 0.60–0.74), and excellent (ICC > 0.74) [12].

All statistical analyses were performed using software MedCalc (version 17.9.7, Medcalc software, Ostend, Belgium), and a p value < 0.05 was considered as statistically significant.

## Results

### Multiple comparison analysis of the DTI parameters in the tibialis anterior muscle

**Fig. 4:**
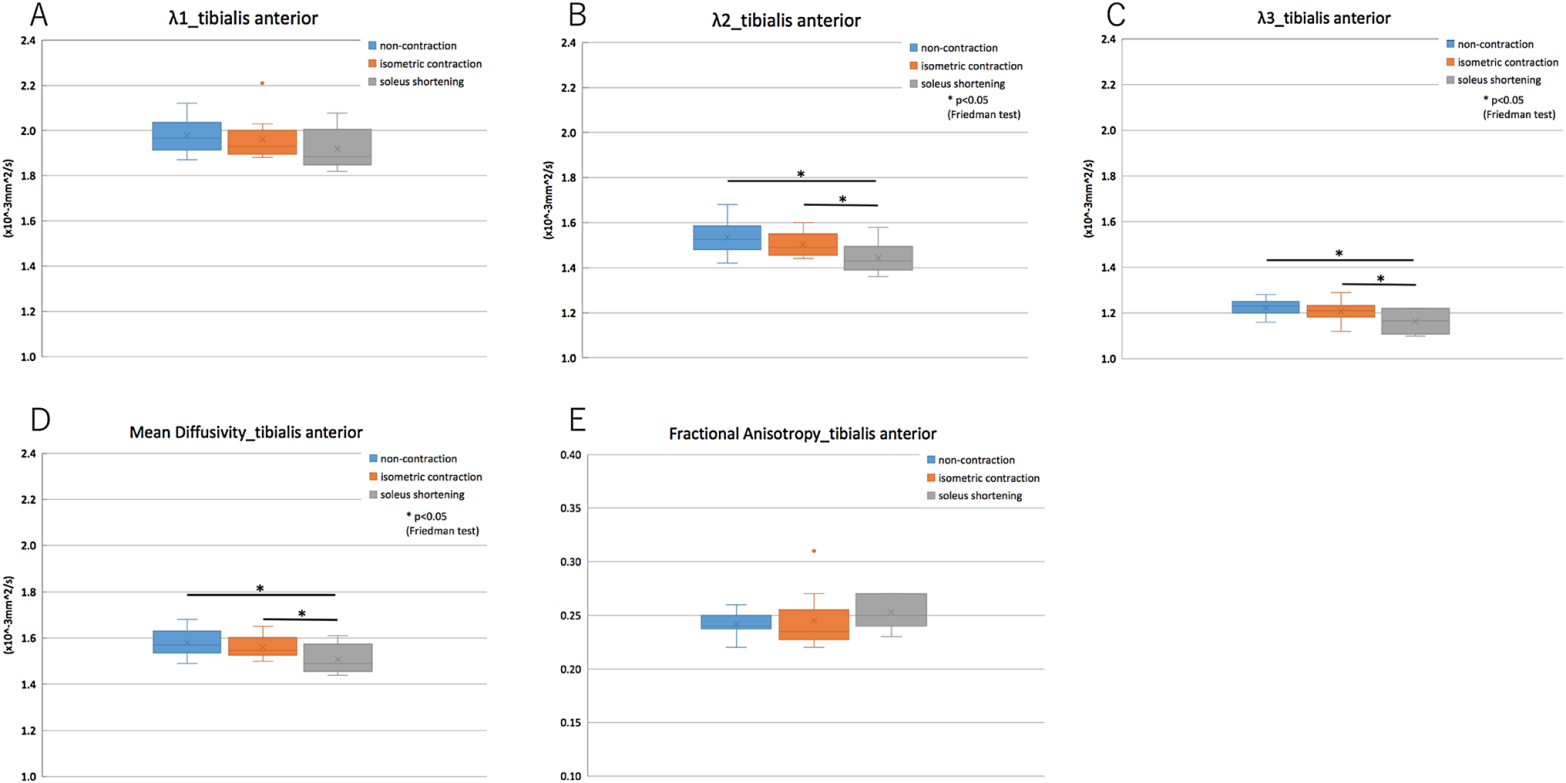
Multiple comparison analysis of DTI values in the tibialis anterior muscle *A* λ_1_, *B* λ_2_, *C* λ_3_, *D* mean diffusivity, and *E* fractional anisotropy The λ_2_, λ_3,_ and MD of the tibialis anterior muscle were significantly lower in soleus shortening compared with that in the non-contraction state and in isometric contraction (*p* < 0.05)

The λ_2_, λ_3,_ and MD were significantly lower in soleus shortening compared with that of the non-contraction state and isometric contraction (*p* < 0.05) (Fig. 4).

### Multiple comparison analysis of DTI parameters in soleus muscle

**Fig. 5:**
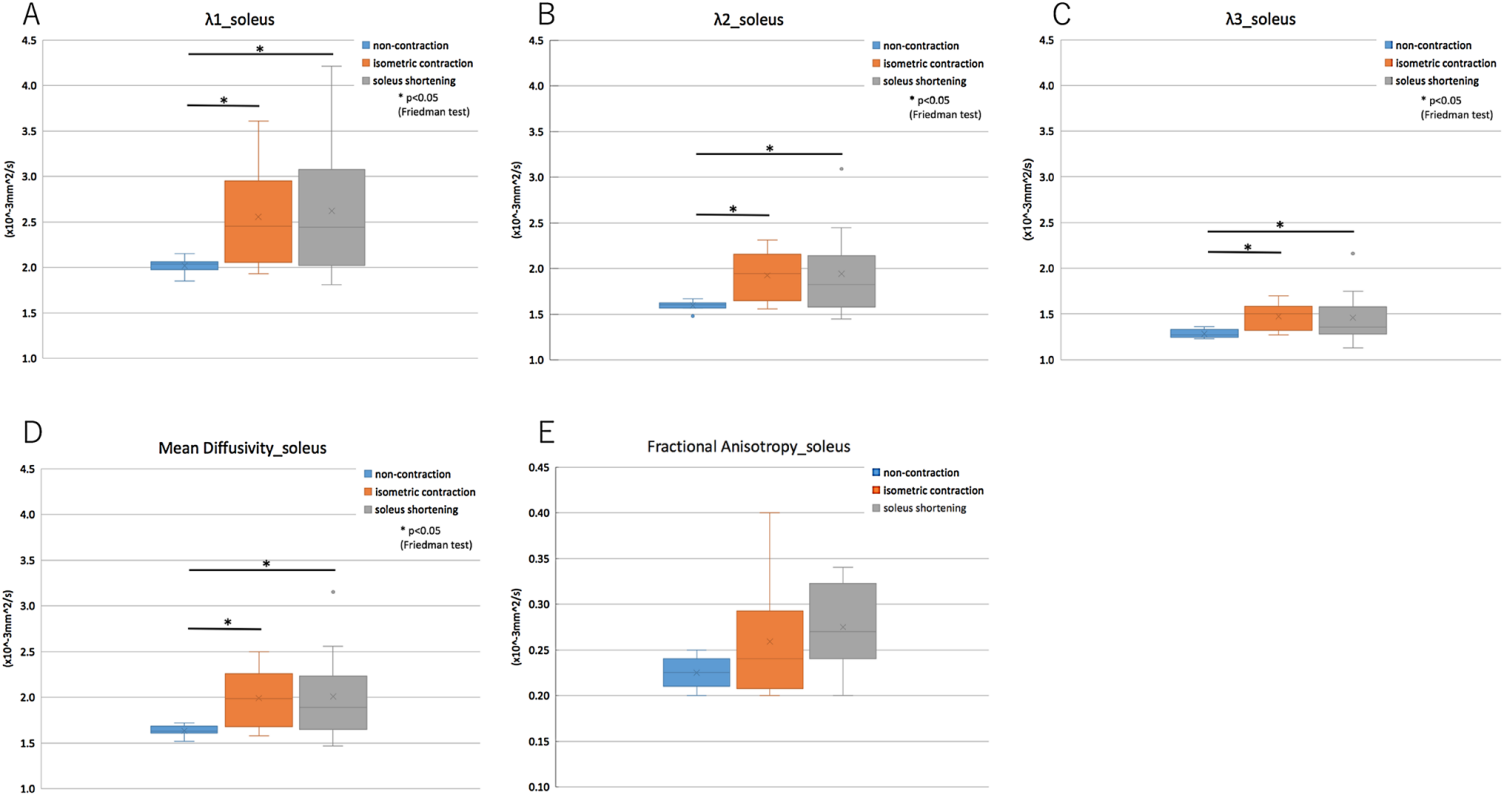
Multiple comparison analysis of DTI values in the soleus muscle *A* λ_1_, *B* λ_2_, *C* λ_3_, *D* mean diffusivity, and *E* fractional anisotropy. The λ_1_, λ_2_, λ_3,_ and MD of the soleus muscle were significantly lower in non-contraction state compared with that in isometric contraction and in soleus shortening (*p* < 0.05)

The λ_1_, λ_2_, λ_3,_ and MD of the soleus muscle were significantly lower in the non-contraction state when compared with that of isometric contraction and soleus shortening (*p* < 0.05) (Fig. 5).

### Correlation between DTI parameters and muscle power measurements

**Fig. 6:**
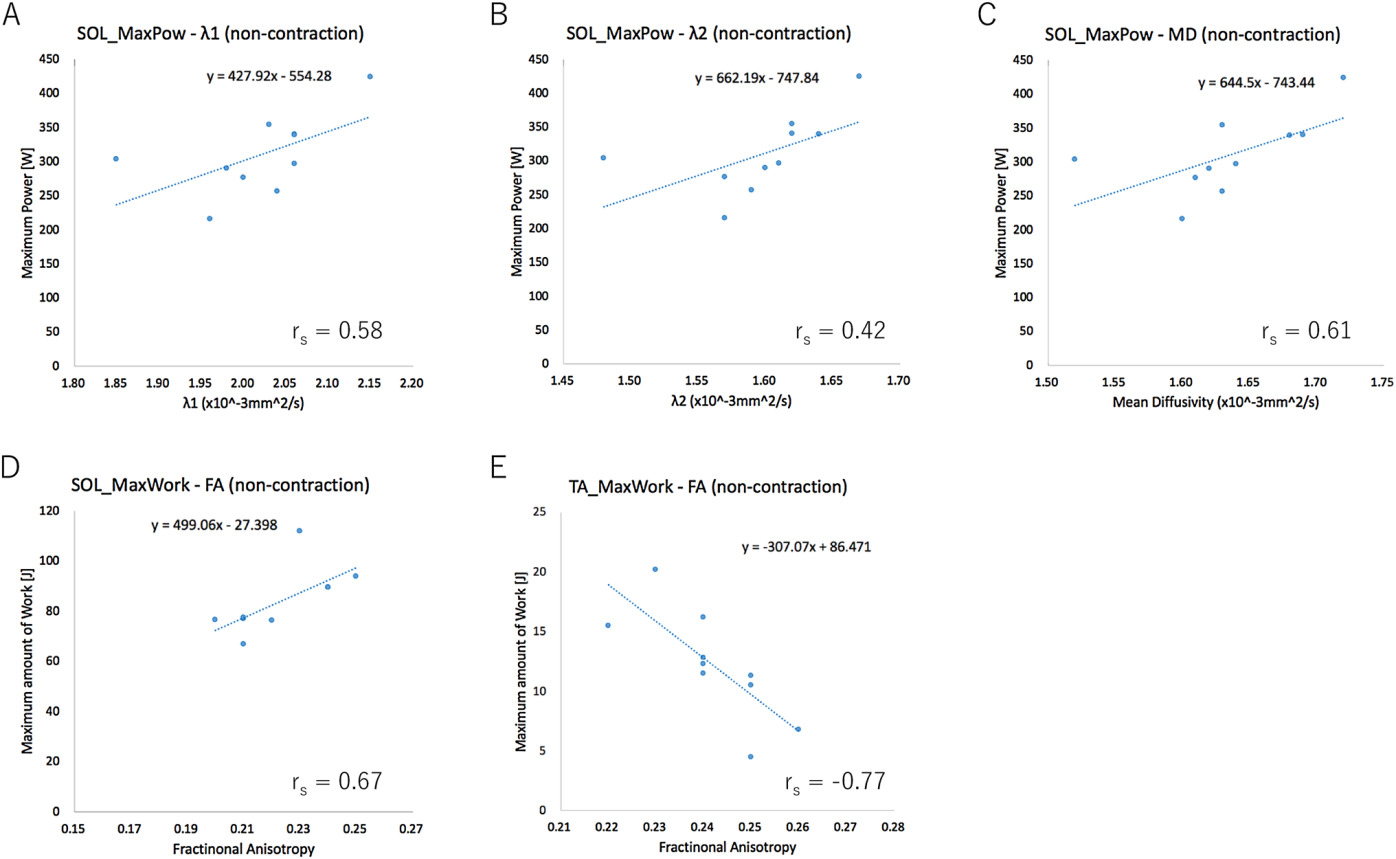
Representative scatter plots of muscle power measurements and DTI parameters Abbreviations: *SOL:* soleus, *TA* tibialis anterior, *MaxPow* maximum power, *MaxWork* maximum amount of work, *MD* mean diffusivity, and *FA* fractional anisotropy. **A** Correlation of the maximum power with the λ_1_ of the soleus muscle in the non-contraction state **B** Correlation of the maximum power with the λ_2_ of the soleus muscle in the non-contraction state **C** Correlation of the maximum power with the MD of the soleus muscle in the non-contraction state **D** Correlation of the maximum amount of work with the FA of the soleus muscle in the non-contraction state **E** Correlation of the maximum amount of work with the FA of the tibialis anterior muscle in the non-contraction state

Table 3 shows the summary of Spearman’s rank correlation coefficients between DTI parameters and muscle power measurements. For the tibialis anterior muscle, a strong positive correlation was found between the maximum amount of work and the λ_1_ with isometric contraction. A strong negative correlation was found between the maximum amount of work and the λ_1_ with soleus shortening, MD with soleus shortening, and FA with the non-contraction state. For the soleus muscle, a strong positive correlation was found between the maximum power and MD in the non-contraction state and between the maximum amount of work and FA in the non-contraction state. Although the correlation strength was moderate or strong with the soleus muscle, the absolute *r*_*s*_ values between the maximum power and the λ_1_, λ_2_, λ_3,_ and MD were higher in the non-contraction state than those in the contraction state. Representative scatter plots are shown in Fig. 6.

**Table 3:** Spearman’s rank correlation coefficient between DTI parameters and muscle power measurements

Spearman’s rank correlation coefficient (r_s_ values)		λ1			λ2			λ3			Mean diffusivity			Fractional anisotropy		
		Non-contraction	Isometric contraction	Soleus shortening	Non-contraction	Isometric contraction	Soleus shortening	Non-contraction	Isometric contraction	Soleus shortening	Non-contraction	Isometric contraction	Soleus shortening	Non-contraction	Isometric contraction	Soleus shortening
Tibialis anterior	Maximum power	0.19 (*p* = 0.608)	0.13 (*p* = 0.731)	− 0.19 (*p* = 0.604)	0.41 (*p* = 0.238)	0.49 (*p* = 0.152)	0.10 (*p* = 0.788)	0.32 (*p* = 0.370)	0.49 (*p* = 0.152)	− 0.01 (*p* = 0.971)	0.31 (*p*=0.382)	0.44 (*p* = 0.205)	− 0.05 (*p* = 0.885)	− 0.32 (*p* = 0.370)	− 0.22 (*p* = 0.549)	− 0.38 (*p* = 0.276)
	Maximum amount of work	− 0.49 (*p* = 0.149)	0.66 (*p* = 0.038)	− 0.61 (*p* = 0.060)	− 0.40 (*p* = 0.249)	0.00 (*p* = 0.982)	− 0.50 (*p* = 0.145)	0.01 (*p* = 0.983)	0.28 (*p* = 0.438)	− 0.55 (*p* = 0.103)	− 0.39 (*p*=0.265)	0.51 (*p* = 0.129)	-0.62 (*p* = 0.057)	− 0.77 (*p* = 0.009)	0.50 (*p* = 0.143)	− 0.22 (*p* = 0.542)
Soleus	Maximum power	0.58 (*p* = 0.076)	0.10 (*p* = 0.786)	0.42 (*p* = 0.225)	0.58 (*p* = 0.076)	0.24 (*p* = 0.505)	0.43 (*p*=0.209)	0.46 (*p* = 0.177)	0.15 (*p* = 0.685)	0.45 (*p* = 0.188)	0.61 (*p*=0.059)	0.13 (*p* = 0.714)	0.44 (*p* = 0.205)	0.04 (*p* = 0.907)	0.11 (*p* = 0.753)	0.36 (*p* = 0.305)
	Maximum amount of work	0.56 (*p* = 0.091)	0.24 (*p* = 0.508)	− 0.38 (*p* = 0.283)	0.42 (*p* = 0.228)	0.22 (*p* = 0.535)	− 0.39 (*p* = 0.266)	− 0.16 (*p* = 0.650)	0.04 (*p* = 0.910)	− 0.42 (*p* = 0.228)	0.35 (*p* = 0.326)	0.20 (*p*=0.572)	− 0.39 (*p* = 0.269)	0.67 (*p* = 0.003)	0.34 (*p* = 0.338)	− 0.17 (*p* = 0.634)

### Correlation between CSA and muscle power measurements, and between CSA and DTI parameters

Spearman rank correlation coefficients (r values) between CSA and muscle power measurements, and between CSA and DTI parameters in the non-contraction state are shown in Table 4. There was a very strong positive correlation between CSA and maximum amount of work in the tibialis anterior muscle, a strong negative correlation between CSA and FA in the tibialis anterior muscle, and a moderate positive correlation between CSA and FA in the soleus muscle. However, the correlations of CSA with eigenvalues and MD were very weak to weak.

**Table 4:** Spearman rank correlation coefficients for CSA with muscle power measurements and with DTI parameters in the non-contraction state

Spearman’s rank correlation coefficient (r_s_ values)	CSA vs muscle power measurements		CSA vs DTI parameters				
	Vs maximum power	Vs maximum amount of work	Vs λ1	Vs λ2	Vs λ3	Vs mean diffusivity	Vs fractional anisotropy
Tibialis anterior	0.36 (*p* = 0.313)	0.81 (*p* = 0.007)	0.06 (*p* = 0.872)	0.02 (*p* = 0.966)	0.23 (*p* = 0.516)	0.05 (*p* = 0.892)	− 0.61 (*p* = 0.063)
Soleus	0.27 (*p* = 0.448)	0.40 (*p* = 0.257)	0.21 (*p* = 0.555)	0.27 (*p* = 0.441)	− 0.35 (*p* = 0.314)	0.22 (*p* = 0.542)	0.57 (*p* = 0.094)

### Intra- and inter-observer reproducibility of DTI parameters in manual segmentation

Overall intra- and inter-ICCs of DTI parameters for the soleus and tibialis anterior muscles are shown in Supplementary Table 1. Intra-observer reproducibility was excellent (soleus muscle: intra-ICC 0.998, inter-ICC 0.997; tibialis anterior muscle: intra-ICC 0.999, inter-ICC 0.999). Intra- and inter-ICCs of each DTI parameter are shown in Supplementary Table 2. Reproducibility of each DTI parameter was excellent, except for inter-observer reproducibility of FA for the tibialis anterior muscle (good, inter-ICC 0.671). Intra- and inter-ICCs of DTI parameters in each position are shown in Supplementary Table 3. Reproducibility of DTI parameters in each position and muscle contraction state were excellent (0.995–1.000).

## Discussion

In this study, the values of the DTI parameters in calf muscles were significantly changed corresponding to ankle position and/or to muscle contraction status. Based on the report of Galban et al., the mechanism for the increase in all three eigenvalues are as follows: increased λ_1_ values may represent the increased diffusivity along the long axis of muscle fibers associated with elongation of the sarcomere, the increased λ_2_ values may represent the increased diffusivity along the sheets of individual muscle fibers within the endomysium during muscle contraction, and the increased λ_3_ may represent the enlargement of each muscle fiber [4]. We found that the changes in the eigenvalues were more significant in the soleus muscle compared with that in the tibialis anterior muscle, because the muscle contraction used for our study was soleus-muscle dominant. Hatakenaka et al. compared the DTI parameters of calf muscles between three ankle joint positions (plantar flexion, intermediate, and dorsiflexion), and found increased λ_2_ and λ_3_ values, and decreased λ_1_ and FA values with plantar flexion [10]. Our results were similar for the λ_2_ and λ_3_ values, but the findings for the λ_1_ and FA values were different. One of the reasons for this discrepancy may be that the soleus muscle has a pennation angle in muscle fiber orientation, and the differences in pennation angles among the subjects and in muscle contraction may affect muscle fiber length, the principal eigenvalue, and FA value. Our study found that the DTI parameters changed during soleus-dominant isometric contraction. When compared to the non-contraction state, the macroscopic lengths of skeletal muscle with isometric contraction were assumed to be similar, however, the microscopic length of muscle fibers may differ. Based on our results, the changes in the values of the DTI parameters may represent the changes in the microscopic structure of skeletal muscle.

Our study found a positive correlation between the maximum power and FA in the soleus muscle in the non-contraction state. In a previous report by Scheel et al., a negative correlation was seen between the maximum torque and FA [5]. The discrepancy between our results and Scheel’s study may be that Scheel’s study was performed with a 90-degree ankle joint position which was different from the angle joint and muscle contraction in our study. In our study on the correlation between the maximum amount of work and FA in the non-contraction state, a positive correlation was found in the soleus muscle and a negative correlation was found in the tibialis anterior muscle. One of the reasons for this discrepancy is the difference in the dominant muscle fiber type between the soleus and tibialis anterior muscles. Skeletal muscle fibers can be categorized as two types based on their main composition and functions: type 1 fibers (slow-twitching fibers, highly resistant to fatigue) and type 2 fibers (fast-twitching fibers, suited for fast and powerful contractions). In general, the predominant muscle fibers of the soleus muscle are type 1 and the tibialis anterior muscles are type 2. Scheel et Al. have reported that the FA values correlated significantly depending on the proportion of fiber types [13].

Among the three different ankle joint angles and muscle contraction states, a correlation between muscle power measurements and DTI values had a tendency to be higher in the non-contraction state. The non-contraction muscle state with a neutral ankle joint angle may be a better patient leg position during DTI acquisition, especially for comparison among patients or for follow-up DTI examination.

In our study, there were positive correlations between CSA of the muscle and muscle power measurements. However, the degree of correlation in maximum amount of work differed between the tibialis anterior and soleus. This result may reflect the difference in composition of muscle fiber between these two muscles. The degree of correlation in maximum power was low for both muscles, which may be due to microscopic muscle quality and extramyocellular structures such as fatty tissue affecting performance, in addition to the muscle volume. The correlation between CSA and FA was strong for the tibialis anterior and moderate for the soleus, which suggests that FA may be a good indicator of early detection of muscle mass changes.

There are some limitations in the study. First, the number of subjects was low and the subjects were limited to young adult males. However, Galban et al. reported that FA and MD of the lower leg muscle differ between males and females [7], and for this reason, only healthy young adult males were enrolled in the study to eliminate the effects of gender on DTI parameters. A future study is required with a larger number of subjects, including females and older adults. Second, we did not evaluate the reproducibility of the DTI acquisition and muscle power measurements. Third, we performed evaluations only at the level of the muscle belly, and not at the muscle insertion. This was because the length of the receiver coil used in the study was not long enough to evaluate both the muscle belly and insertion.

In conclusion, DTI values changed significantly depending on both the ankle joint position and muscle contraction state. For comparison or follow-up DTI examination of skeletal muscle, both a uniform joint position and muscle contraction state should be used. Because DTI can be scanned in several minutes, it can be added to routine clinical MRI protocols. A protocol including DTI may permit evaluation of skeletal muscle performance of athletes, analysis of the effects of rehabilitation after trauma or skeletal muscle disease, and morphological evaluation.

## Supplementary Information

Below is the link to the electronic supplementary material.

Supplementary file1 Table 1 Intra- and inter-ICCs of DTI parameters for the soleus and tibialis anterior muscles. Table 2 Intra- and inter-ICCs for DTI parameters. Table 3 Intra- and inter-ICCs for DTI parameters for each position. (PPTX 49 KB)
