# Correction to: Diffusion tensor imaging (DTI) of human lower leg muscles: correlation between DTI parameters and muscle power with different ankle positions

**DOI:** 10.1007/s11604-022-01287-w

**Published:** 2022-05-16

**Authors:** Shoichiro Takao, Maho Kaneda, Mihoko Sasahara, Suzuka Takayama, Yoshitaka Matsumura, Tetsuya Okahisa, Tsuyoshi Goto, Nori Sato, Shinsuke Katoh, Masafumi Harada, Junji Ueno

**Affiliations:** 1 Department of Diagnostic Radiology, Graduate School of Health Sciences, Tokushima University, 3-18-15, Kuramoto-Cho, Tokushima, 770-8509 Japan; 2 Department of Radiology, Grandsoul Nara, Nara, Japan; 3 Department of Radiology, Tokushima University, Tokushima, Japan; 4 Department of Radiology, Rakuwakai Otowa Hospital, Kyoto, Japan; 5 Department of Radiological Technology, Tokushima Bunri University, Kagawa, Japan; 6 Graduate School of Health Sciences, Tokushima University, Tokushima, Japan; 7 Rehabilitation Center, Tokushima University Hospital, Tokushima, Japan; 8 Department of Rehabilitation, Japanese Red Cross Tokushima Hinomine Rehabilitation Center, Tokushima, Japan; 9 Department of Radiology, Kitajima Taoka Hospital, Tokushima, Japan

## Correction to: Japanese Journal of Radiology 10.1007/s11604-022-01274-1

In the original publication, Table 2 has been published incompletely. The complete version of Table 2 is given in this Correction.

**Table 2:** Types of muscle contraction

	non-contraction	isometric contraction	soleus (SOL) shortening
			
angle of ankle joint	neutral position	neutral position	maximum plantar flexion
status of tibialis anterior muscle	non-contraction	isometric contraction	elongation
status of soleus muscle	non-contraction	isometric contraction	contraction with shortening

The original publication has been corrected.

