# Supplementary material for: Diffusion tensor imaging (DTI) of human lower leg muscles: correlation between DTI parameters and muscle power with different ankle positions
Source: Jpn J Radiol. 2022 Apr 9;40(9):939–48. doi: 10.1007/s11604-022-01274-1 (PMC9441424; doi:10.1007/s11604-022-01274-1)
Supplement: Supplementary file 1 — Supplementary file1 Table 1 Intra- and inter-ICCs of DTI parameters for the soleus and tibialis anterior muscles. Table 2 Intra- and inter-ICCs for DTI parameters. Table 3 Intra- and inter-ICCs for DTI parameters for each position. (PPTX 49 KB) [file 11604_2022_1274_MOESM1_ESM.pptx]

## Slide 1
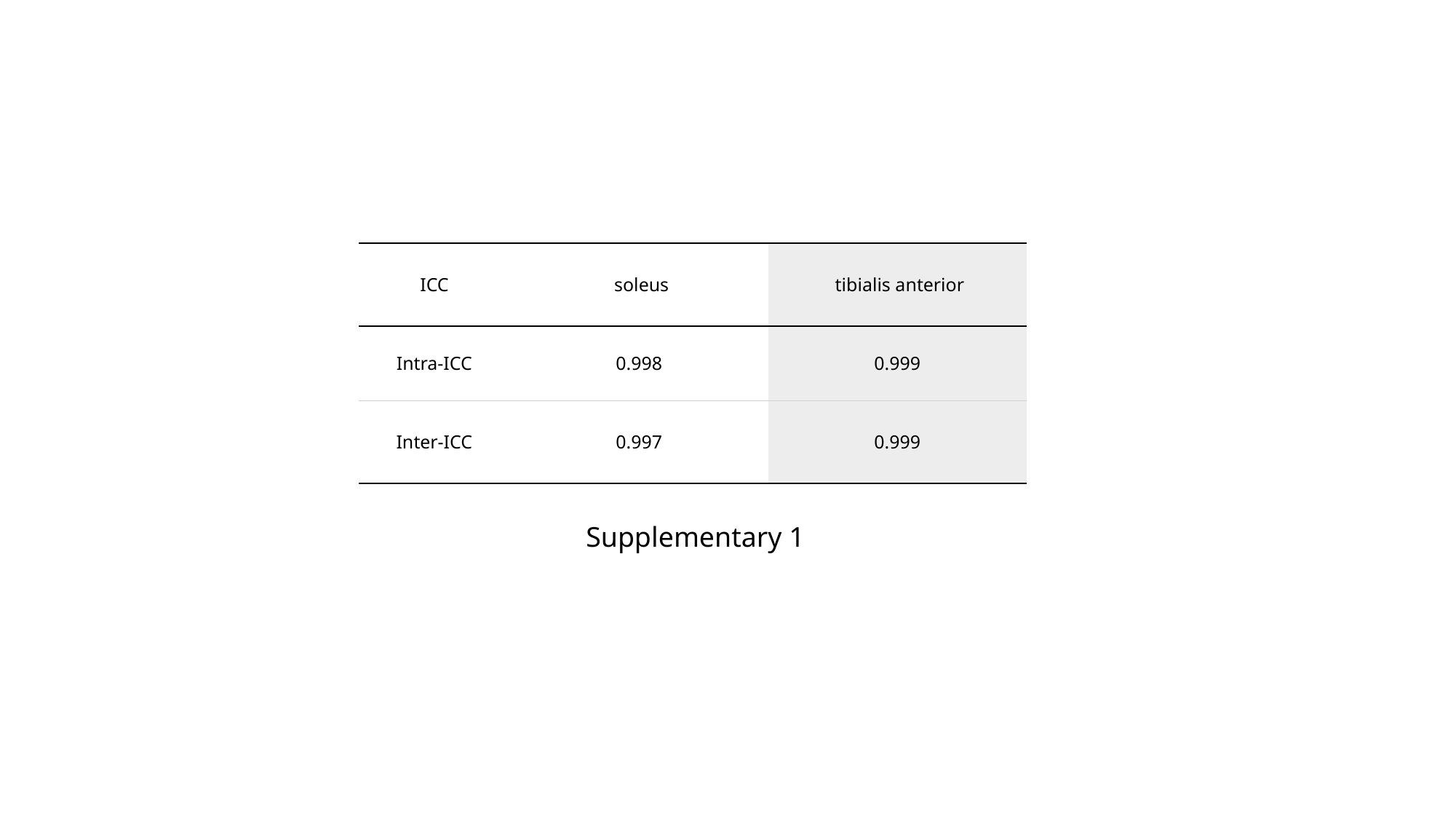

| ICC | soleus | tibialis anterior |
| --- | --- | --- |
| Intra-ICC | 0.998 | 0.999 |
| Inter-ICC | 0.997 | 0.999 |
Supplementary 1

## Slide 2
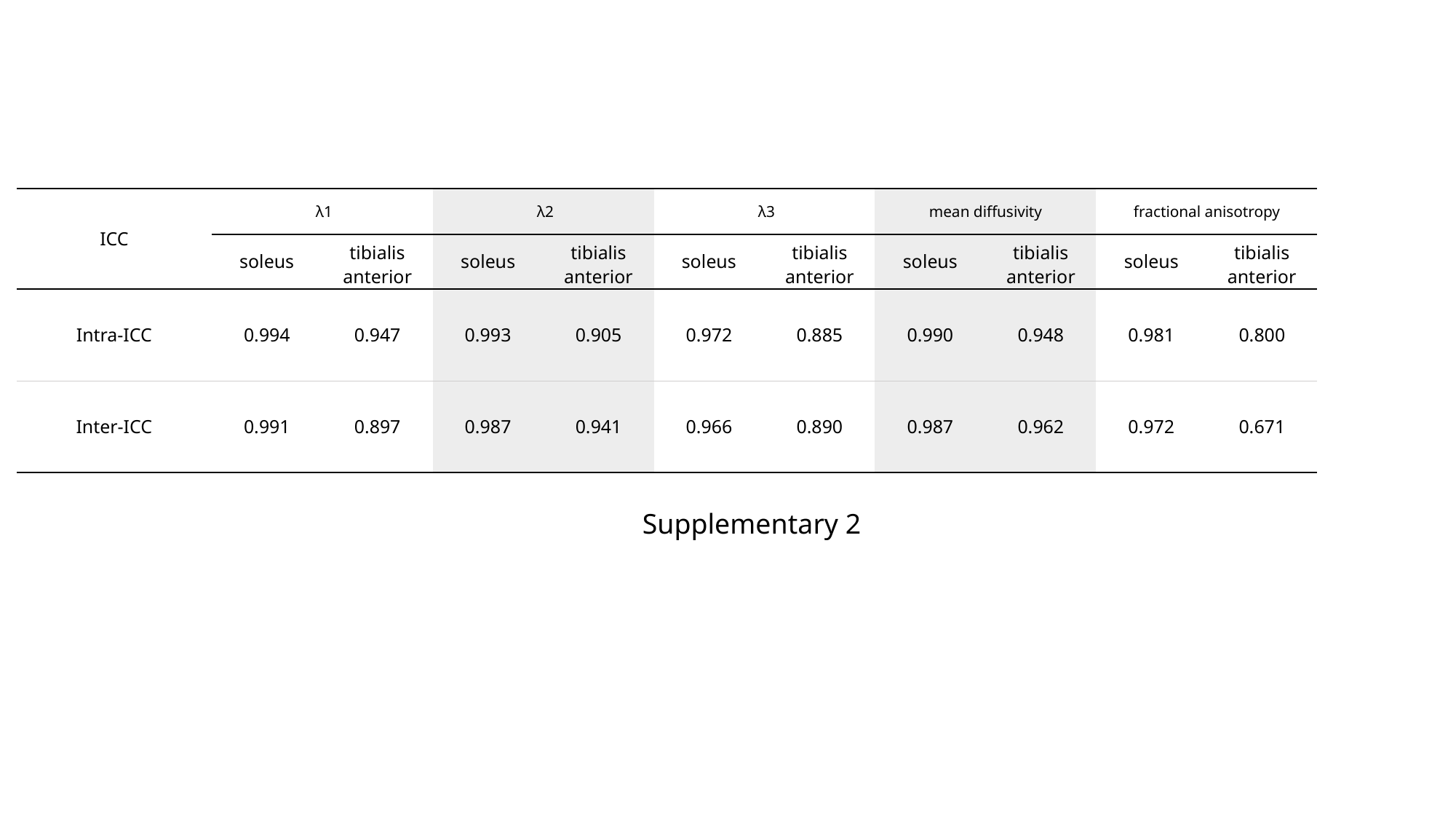

| ICC | λ1 | | λ2 | | λ3 | | mean diffusivity | | fractional anisotropy | |
| --- | --- | --- | --- | --- | --- | --- | --- | --- | --- | --- |
| | soleus | tibialis anterior | soleus | tibialis anterior | soleus | tibialis anterior | soleus | tibialis anterior | soleus | tibialis anterior |
| Intra-ICC | 0.994 | 0.947 | 0.993 | 0.905 | 0.972 | 0.885 | 0.990 | 0.948 | 0.981 | 0.800 |
| Inter-ICC | 0.991 | 0.897 | 0.987 | 0.941 | 0.966 | 0.890 | 0.987 | 0.962 | 0.972 | 0.671 |
Supplementary 2

## Slide 3
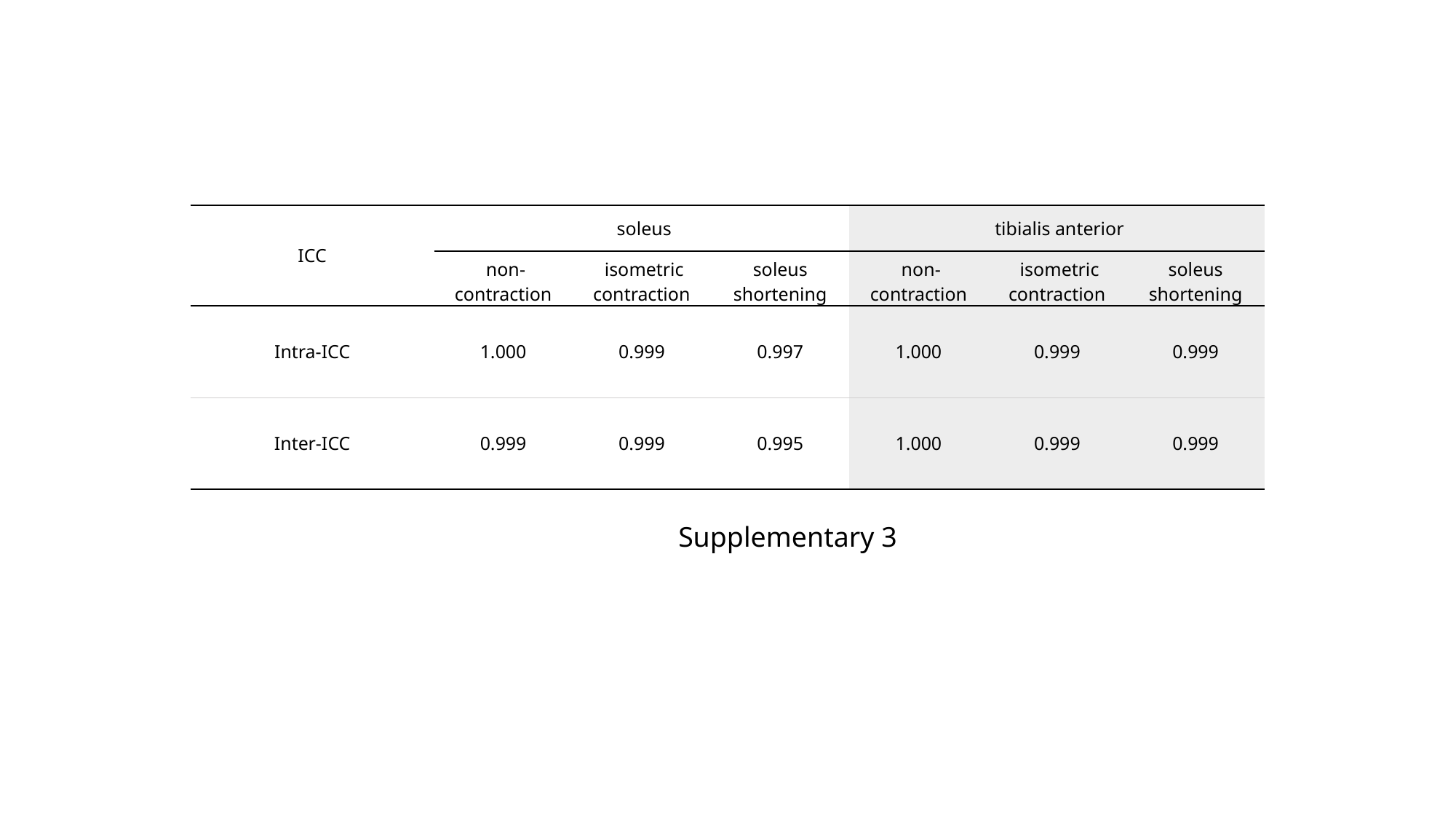

| ICC | soleus | | | tibialis anterior | | |
| --- | --- | --- | --- | --- | --- | --- |
| | non- contraction | isometric contraction | soleus shortening | non- contraction | isometric contraction | soleus shortening |
| Intra-ICC | 1.000 | 0.999 | 0.997 | 1.000 | 0.999 | 0.999 |
| Inter-ICC | 0.999 | 0.999 | 0.995 | 1.000 | 0.999 | 0.999 |
Supplementary 3
